# Gegen Qinlian Decoction Ameliorates Hyperuricemia-Induced Renal Tubular Injury via Blocking the Inflammatory Signaling Pathway

**DOI:** 10.3389/fphar.2021.665398

**Published:** 2021-05-04

**Authors:** Xiao-Jun Wang, Yi-Ding Qi, Hao-Chen Guan, Hua-Gang Lin, Pei-Qing He, Kang-Wei Guan, Lei Fu, Mao-Qing Ye, Jing Xiao, Tao Wu

**Affiliations:** ^1^ Department of Traditional Chinese Medicine, Huadong Hospital Affiliated to Fudan University, Shanghai, China; ^2^ Shanghai Key Laboratory of Clinical Geriatric Medicine, Huadong Hospital Affiliated to Fudan University, Shanghai, China; ^3^ Department of Cardiology, Huadong Hospital Affiliated to Fudan University, Shanghai, China; ^4^ Department of Nephrology, Shanghai General Hospital, Shanghai Jiao Tong University School of Medicine, Shanghai, China; ^5^ Department of Nephrology, Huadong Hospital Affiliated to Fudan University, Shanghai, China

**Keywords:** Gegen Qinlian decoction, hyperuricemia, NLRP3, apoptosis, pharmacological network analysis

## Abstract

**Background:** Gegen Qinlian decoction (GGQLD) is a typical traditional Chinese medicine (TCM) prescription documented in *Shang Han Lun*. Clinically, GGQLD has been utilized to manage the inflammatory symptoms of metabolic diseases and to protect against renal damage in China. In the present study, a hypothesis was proposed that the multi-target solution of GGQLD produced anti-inflammatory effects on ameliorating hyperuricemia (HUA).

**Methods:** A total of 30 primary HUA patients receiving GGQLD treatment (two doses daily) for 4 weeks were selected. Then, differences in uric acid (UA) levels and expression of peripheral blood mononuclear cells (PBMCs) and urinary exosomes before and after treatment were analyzed. The therapeutic indexes for the active ingredients in GGQLD against HUA were confirmed through pharmacological subnetwork analysis. Besides, the HUA rat model was established through oral gavage of potassium oxonate and treated with oral GGQLD. In addition, proximal tubular epithelial cells (PTECs) were stimulated by UA and intervened with GGQLD for 48 h. Subsequently, RNA-seq, flow cytometry, and confocal immunofluorescence microscopy were further conducted to characterize the differences in UA-mediated inflammation and apoptosis of human renal tubular epithelial cells pre- and post-administration of GGQLD. In the meanwhile, quantitative real-time PCR (qPCR) was carried out to determine gene expression, whereas a western blotting (WB) assay was conducted to measure protein expression.

**Results:** Our network analysis revealed that GGQLD treated HUA via the anti-inflammatory and antiapoptotic pathways. Additionally, NLPR3 expression significantly decreased in PBMCs and urinary exosomes of HUA patients after GGQLD treatment. *In vivo*, GGQLD treatment alleviated HUA-induced renal inflammation, which was associated with decreased expression of NLRP3 inflammasomes and apoptosis-related mRNAs. Moreover, GGQLD promoted renal UA excretion by inhibiting the activation of GSDMD-dependent pyroptosis induced by NLRP3 inflammasomes and by reducing apoptosis via the mitochondrial apoptosis signaling pathway *in vitro*.

**Conclusion:** This study indicates that GGQLD efficiently reduces inflammatory responses while promoting UA excretion in HUA. Our findings also provide compelling evidence supporting the idea that GGQLD protects against the UA-mediated renal tubular epithelial cell inflammation through the mitochondrial apoptosis signaling pathways. Taken together, these findings have demonstrated a novel therapeutic method for the treatment of HUA.

## Introduction

Hyperuricemia (HUA) is a factor that independently predicts the risk of kidney diseases (Prasad and Qing, 2015; Srivastava et al., 2018; Tsai et al., 2018). Generally, HUA is manifested as macrophage infiltration, tubular damage, and upregulated inflammatory mediator levels (Zhou et al., 2012; Xiao et al., 2018; Braga et al., 2020). The renal proximal tubule plays a pivotal role in transporting renal urate, which is a major site of urate reabsorption (Lipkowitz, 2012) and exerts a remarkable role in HUA occurrence and development. In our previous study, soluble uric acid (UA) triggers NLRP3 inflammasome production, IL-1β expression, and caspase-1 activation in human PTECs, which could thus induce the secretion of pro-inflammatory cytokines and activate the innate immunity (Xiao et al., 2015; Xiao et al., 2016). Therefore, it is of importance to develop a treatment against HUA to prevent renal tubular injury in the future. Till the present, numerous patients have experienced a relapse after the withdrawal of anti-hyperuricemic drugs such as benzbromarone.

For thousands of years, combination therapy has been promoted in traditional Chinese medicine (TCM) to treat different disorders. Among them, Gegen Qinlian decoction (GGQLD), one of the typical TCM prescriptions, was originally documented in the *Treatise on Exogenous Febrile Disease* in the Han Dynasty (202 BC–220 CE). GGQLD comprises *Pueraria montana* var. *lobata* (Gegen), *Scutellaria baicalensis Georgi* (Huangqin), *Glycyrrhiza uralensis Fisch. ex DC* (Gancao), and *Coptis chinensis Franch* (Huanglian). It has been extensively utilized for the clinical treatment of gastrointestinal diseases for approximately 2,000 years, in particular for damp-heat syndrome–related diarrhea (Bi et al., 2013; Tian et al., 2016). Moreover, the clinical efficacy of GGQLD in treating ulcerative colitis (UC) has been verified (Zhao et al., 2020). Experiments *in vitro* and *in vivo* suggest that some active ingredients in GGQLD, like berberine, baicalin, and puerarin, obviously mitigate oxidative stress (OS) and inflammation (Dinesh and Rasool, 2017; Guarino et al., 2017; Yu et al., 2021). We previously discovered that GGQLD exerted diabetes-mitigating (Xu et al., 2011; Li et al., 2013; Zhang et al., 2013) and anti-inflammation effects through its active ingredients (Tian et al., 2013; Ryuk et al., 2017; Wu et al., 2019). Besides, inflammatory response, lipid, and glucose metabolic disorders are also important for HUA. Therefore, guided by the TCM theory of “treating different diseases with the same therapy,” GGQLD can be sometimes used to treat HUA. However, the underlying mechanism of action has not been clarified.

By network pharmacology, we discovered that GGQLD might treat HUA via the anti-inflammatory and antiapoptotic pathways. Further experiments showed that GGQLD alleviated the inflammatory state of HUA patients and improved renal inflammation in HUA rats. Moreover, *in vitro* experiment confirmed that GGQLD played a role in the inflammation and apoptosis of PTECs induced by UA.

## Materials and Methods

### Materials and Reagents

Cell culture medium and human primary renal PTECs were provided by ScienCell (San Diego, CA, United States). Anti-SLC2A9 (URAT1) antibodies, TSG101, CD63, caspase-1, GSDMD, IL-1β, caspase-3, caspase-8, caspase-9, cytochrome c, Bcl-2, and Bax were purchased from ABclonal (Wuhan, China). Oxonic acid (OA) and UA were provided by Sigma (St. Louis, MO, United States). CIAS1/NALP3, GAPDH, and anti-GLUT9 were provided by Abcam (Cambridge, United Kingdom). In addition, the CCK-8 assay kit was provided by Jiwei Biological Technology (Shanghai, China).

### Preparation of GGQLD

GGQLD, a famous decoction documented in the *Treatise on Exogenous Febrile Disease* around 1,900 years ago, was used and approved by the Huadong Hospital Affiliated to Fudan University. The dried herbs, including *Pueraria montana* var. *lobata* (Gegen), *Scutellaria baicalensis Georgi* (Huangqin), *Glycyrrhiza uralensis Fisch. ex DC* (Gancao), and *Coptis chinensis Franch* (Huanglian), in the ratio of 8:3:3:2 (w/w/w/w), were first soaked in distilled water with 15-fold volumes of herbs (v/w) for 30 min and then extracted by decoction twice, 2 h for the first time and 2 h for the second time, with 8-fold volumes of water to herbs (v/w). After filtration, the solution was evaporated under reduced pressure to a suspension with a final density of 1 g/ml and stored at 4°C for further use (Xie et al., 2006).

### High-Performance Liquid Chromatography (HPLC)

Puerarin, baicalin, liquiritin, and berberine (all purities >98%) were provided by Shanghai Yuanzhi Biotechnology Co., Ltd. (Shanghai, China). Supplementary Figure S1 presents the chemical structures of the ingredients. Acetonitrile, formic acid, and methanol of HPLC grade were provided by Merck Company (Darmstadt, Germany). The Milli-Q system (Millipore, Milford, MA, United States) was utilized to obtain deionized water. Other reagents of analytical grade were acquired from commercial sources. To identify the components of GGQLD, we performed HPLC analysis using an Agilent 1260 Infinity brand chromatographic chain with a C18 chromatographic column (250 × 4:6 mm, 5 μm, Thermo Fisher Scientific, NY, United States), along with a column oven, a binary pump, a diode array detector, and an autosampler (Agilent 1100 series). Thereafter, 20 ml ethanol extract was added to the column. Throughout the whole chromatographic analysis process, the column injector was maintained at 25°C. The mobile phase comprised the ammonium formate solution (10 mmol/l, pH 3.0, A) and acetonitrile. For the components, their detection wavelength was set at 270 nm. Subsequently, we identified each compound according to the pure standard absorbance spectra and retention time (Guo et al., 2019).

### Systemic Pharmacological Analysis of GGQLD

We searched the active ingredients of Gegen, Huangqin, Huanglian, and Gancao in GGQLD against the TCMSP database (http://tcmspw.com/index.php) by the thresholds of druglikeness (DL, also known as the similarity between the known medicines and the components) ≥0.18 and oral bioavailability (OB, medicine component oral availability) ≥30%. Based on the TCMSP database (Yao et al., 2013; Liu et al., 2020), we aligned the GGQLD active ingredients with the candidate targets separately, then retrieved targets of diverse origins against the UniProt database (http://www.uniport.org), and acquired the official target gene symbols to perform subsequent assays. A total of 196 HUA-associated targets were identified from the DisGeNET (http://www.disgenet.org) and NCBI databases. Cytoscape 3.8.0 software is the openly accessible bioinformatics platform used to visualize the molecular interaction networks (Deng et al., 2020). In the current study, we adopted Cytoscape to construct the active ingredient–target–disease–medicine interaction network. GO functional annotations and KEGG pathway analyses on the critical therapeutic targets in GGQLD against HUA were analyzed by adopting the DAVID 6.8 database (Komada et al., 2018).

### Participants and Setting

From January 1st, 2020 to October 31st, 2020, a total of 30 male patients with asymptomatic hyperuricemia were enrolled from the Inpatient and Outpatient Departments of the Huadong Hospital Affiliated to Fudan University (Shanghai, China). The patient inclusion criteria are shown below, those aged 18–75 years with a diagnosis of HUA (serum uric acid, SUA > 420 μmol/l). For patients receiving anti-HUA treatment, they should receive washout for 2 weeks, and we only enrolled patients with SUA > 420 μmol/l post-washout. The patient exclusion criteria were as follows: 1) those with an allergic physique or previous allergic history to benzbromarone or TCM, 2) serum creatinine >1.5 mg/dl, 3) two-fold elevation of ALT compared with the normal upper limit, 4) serious stiffness or deformity due to gouty arthropathy, 5) clinically significant arrhythmia, and 6) alcohol abuse history. In addition, patients confirming to any one of the following conditions were also excluded: 1) those having serious concurrent diseases in the hematopoietic system, liver, cerebrovascular system, or kidney, mental diseases, or malignant cancers; 2) those taking salicylate or aspirin (>325 mg/d)-containing medications; 3) those taking hypouricemic medications, 6-mercaptopurine, or azathioprine; and 4) those who had been involved in additional clinical trials in the last 3 months.

In this study, 30 patients were given GGQLD treatment (two doses daily for 4 weeks). SUA and urine uric acid (UUA) were adopted for evaluating the therapeutic efficacy (Table 1). The Local Ethical Committee of Huadong Hospital Affiliated to Fudan University approved our study protocols (No. 20190037).

**TABLE 1:** Clinical data of HUA patients before and after GGQLD treatment.

	Before treatment with GGQLD Mean ± SD	After treatment with GGQLD Mean ± SD	*p* value^#^
SUA	497.70 ± 39.47	418.93 ± 69.64	<0.001
UUA	424.81 ± 157.10	641.62 ± 272.83	0.0128
Scr	85.84 ± 14.28	83.88 ± 12.34	0.3699
BUN	5.37 ± 1.28	5.84 ± 2.42	0.3518
eGFR	85.38 ± 17.35	87.33 ± 13.34	0.4198
TG	2.22 ± 1.08	1.81 ± 0.69	0.0263
TC	4.59 ± 1.04	4.34 ± 0.95	0.1458
IL1β	9.13 ± 1.06	4.67 ± 1.15	0.0005
IL6	7.43 ± 5.80	2.35 ± 0.70	0.0389
IL8	20.58 ± 13.35	6.40 ± 0.77	0.0206
ALT	35.22 ± 33.16	24.96 ± 17.38	0.0380
AST	26.46 ± 15.28	20.87 ± 9.02	0.1043

### Peripheral Blood Mononuclear Cell Isolation by Ficoll-Paque Density Gradient Centrifugation

The Local Ethical Committee of Huadong Hospital Affiliated to Fudan University approved our experimental protocols on human blood (No. 20190037). To be specific, whole blood was centrifuged at 500 × g for 5 min at room temperature to separate the plasma. Thereafter, the equivalent amount of 1× PBS (under ambient temperature) was used to dilute the rest blood samples, followed by Ficoll-Paque underlaying (under ambient temperature) and 30 min of centrifugation (2,000 rpm, 21°C) using a Heraeus Multifuge X3R (Thermo Fisher Scientific), with the deceleration and acceleration being set at 0 and 5, respectively. Afterward, we obtained PBMCs from the interface between plasma and Ficoll-Paque in the 15 ml tube and used 1× PBS to wash the cells twice for 10 min (1,500 rpm, 4°C).

### Purification and Characterization of Urine Exosomes and Pellets

We collected urinary exosomes from 8 controls and 8 GGQLD-intervened HUA patients. Later, 40 ml freshly prepared urine samples were collected to obtain urinary cell pellets. Thereafter, the supernatants were subjected to 30 min of centrifugation at 12,000 × g, followed by standing to isolate the urinary exosomes. ExoQuick-TC for tissue culture media and urine (Exiqon, Woburn, MA, United States) was used to isolate urinary exosomes in accordance with specific protocols. To validate the protocols for exosome purification, we performed cryo-transmission electron microscopy to analyze the size, shape, and morphology of urinary exosomes. The isolation of exosomes was characterized by WB using NanoSight.

### Animal Model and Measurement

A total of 15 male SPF SD rats (age, 8 weeks; weight, 200–250 g) were raised at the Animal Center of Shanghai Rat and Mouse Biotech Co., Ltd. Then, all animals were randomized into three groups, including the 1) Cont, 2) OA (8-week administration of OA at 750 mg/kg/day), and 3) OA + GGQLD (8-week administration of OA at 750 mg/kg/day + 4-week administration of GGQLD at 10 ml/kg/day initiating in week 5) groups, with five in every group. At the end of week 8, each animal was terminated. The 24-h urine was sampled from animals in metabolic cages. Subsequently, the levels of sUA, uUA, serum creatinine (Scr), blood urea nitrogen (BUN), and urinary creatinine (Ucr) were measured through the enzymatic colorimetric assay using a fully automatic chemistry analyzer (MODULAR D/P, Roche). Afterward, the freshly prepared renal cortical samples were obtained immediately after the animals were terminated to measure UA in accordance with specific protocols. Part of the tissues was stained with hematoxylin–eosin (HE) or Masson’s trichrome stain for light microscopy. Next, the dissected tissue samples were preserved at −80°C to carry out immunoblotting and RT-PCR assays. Each animal experiment was approved by the Animal Care and Use Committee of Shanghai Rat and Mouse Biotech Co., Ltd. following guidelines of the National Institutes of Health (NIH Pub. No. 85-23, revised 1996).

### Cell Culture

The epithelial cell medium, which was supplemented with basal medium (500 ml), fetal bovine serum (10 ml, FBS), penicillin/streptomycin (5 ml), and epithelial cell growth supplement (5 ml), was used to culture human primary PTECs. Then, we incubated cells under 37°C and 5% CO_2_ conditions. A 24-h “growth arrest” period was observed within the serum-free medium before stimulation in each experiment.

### Soluble UA Preparation

Previously, we dissolved UA into 1M NaOH to the final concentration of 50 mg/ml (Xiao et al., 2015). Then, we examined the solution so as to ensure that there was no mycoplasma and also filtered the solution (pore size, 22 μm) prior to use. No detectable crystal was observed under polarizing microscopy or in the process of cell incubation.

### Cell Viability Assay on Cells Treated With Different Doses of GGQLD

A Cell Counting Kit-8 (CCK-8, Beijing Solarbio Science and Technology Co., Ltd.) assay was conducted to assess cell viability after GGQLD treatments at diverse doses. Cells (1 × 104 cells/well) were cultivated within the 96-well plate for 24–48 h. The microplate reader was used to measure absorbance (OD) at 450 nm. All experiments were conducted thrice.

### RNA Sequencing

After GGQLD treatment, we harvested PTECs and washed them twice with ice-cold PBS. We collected six samples altogether, among which three were from the GGQLD group while three were from the control group. Trizol reagent (Takara, Dalian, China) was employed to extract total RNA in accordance with specific protocols. Libraries were constructed and mRNA was sequenced by Nuohe Zhiyuan Technology Company (Beijing, China). In addition, DESeq (version 1.30.0) was utilized to identify differentially expressed genes (DEGs). The false discovery rate (FDR) was applied to adjust p value, and the significance thresholds of fold change (FC) ≥ 2 and p < 0.05 were applied.

### Annexin V-FITC/PI Double-Labeled Flow Cytometry

Flow cytometric analysis was conducted with an Annexin V-FITC apoptosis detection kit (DOJINDO; Kyushu, Japan) to analyze cell apoptosis under specific instructions. After UA and GGQLD treatments, PTECs were harvested and rinsed with ice-cold PBS, followed by staining using the binding buffer that contained Annexin V-FITC/PI at 4°C for 15 min in the dark. Finally, flow cytometric analysis (Beckman Coulter, Fullerton, CA, United States) was performed to record cells.

### Confocal Immunofluorescence Microscopy

Cells were cultured onto a laser confocal cell culture dish (Thermo Fisher Scientific, NY, United States) in accordance with specific protocols. 24 h later, the cells were washed with PBS and fixed by 4% paraformaldehyde in PBS. Thereafter, 10% BSA contained within PBS was utilized to block the cells for 30 min, whereas the GSDMD and NLRP3 antibodies in 10% BSA were used to incubate the cells at 4°C overnight. Then, the cells were rinsed with PBS thrice and incubated with goat anti-rabbit antibodies (Invitrogen, Grand Island, NY, United States) for 1 h in the dark. The cells were washed consecutively thrice, and a confocal imaging system (LSM 780;Carl Zeiss, Jena, Germany) was employed to examine the dishes.

### RNA Extraction and Quantitative Real-Time PCR

Trizol (Life Technologies, United States) was employed to isolate total cellular and tissue RNA in accordance with specific protocols. The content and purity of RNA were detected through a NanoDrop 2000 (Thermo Fisher Scientific, United States). To carry out mRNA quantification, we applied a reverse transcription kit (Vazyme, China) for the reverse transcription of total RNA according to specific instructions. Afterward, SYBR Green Master Mix (Vazyme, China) was utilized to perform qPCR, whereas a Roche Light Cycler system (Roche, Switzerland) was adopted for analysis, with GAPDH being an endogenous control. Table 2 lists the primer sequences. Gene expression was normalized to the GAPDH level, which was presented in the manner of FC (2−ΔΔCT). All results were repeated thrice.

**TABLE 2:** Primers for real-time PCR.

Gene	Forward primer	Reverse primer
NLRP3	GCA​GCG​ATC​AAC​AGG​CGA​GAC	TCC​CAG​CAA​ACC​TAT​CCA​CTC​CTC
IL1β	CTC​CAC​CTC​CAG​GGA​CAG​GAT​ATG	TCA​TCT​TTC​AAC​ACG​CAG​GAC​AGG
IL18	GCT​GCT​GAA​CCA​GTA​GAA​GAC​A	TGC​CAA​AGT​AAT​CTG​ATT​CCA​GGT
TNF	TGG​CGT​GGA​GCT​GAG​AGA​TAA​CC	CGA​TGC​GGC​TGA​TGG​TGT​GG
Bax	GAC​GCA​TCC​ACC​AAG​AAG​CTG​AG	GCT​GCC​ACA​CGG​AAG​AAG​ACC
Bcl2	GGG​CTA​CGA​GTG​GGA​TAC​TGG​AG	TCG​GTT​GCT​CTC​AGG​CTG​GAA​G
CYCS	AAA​GGG​AGG​CAA​GCA​CAA​GAC​TG	ATT​GGC​GGC​TGT​GTA​AGA​GTA​TCC
CASP8	TCT​ACG​GAA​CGG​ATG​GGA​AGG​AAG	CAG​GCA​CAG​GCA​CCG​CTT​TC
CASP3	GTG​GAG​GCC​GAC​TTC​TTG​TAT​GC	TGG​CAC​AAA​GCG​ACT​GGA​TGA​AC

### WB Analysis of the Cultured Human PTECs

Supernatants of cell culture were collected according to the previous description and then the lysis buffer supplemented with protease inhibitor cocktails (Sigma, St Louis, MO, United States) was used to lyse the rest cells. After collecting total proteins, we carried out WB analysis according to the previous description (Xiao et al., 2015). In the present work, the following primary antibodies (all were diluted at 1:1,000) were adopted, including rabbit pAb Glut9, rabbit pAb URAT1, rabbit pAb caspase-1, rabbit pAb caspase-3, rabbit pAb NLRP3, rabbit pAb IL-1β, rabbit pAb GSDMD, rabbit pAb Bax, rabbit pAb caspase-8, rabbit pAb cytochrome c, rabbit pAb Bcl-2, and rabbit pAb caspase-9.

### Statistical Analysis

Data were presented in the manner of means ± SD, except as otherwise noted. SPSS19.0 (SPSS, Inc., Chicago, IL, United States) was applied for all statistical analyses. For continuous variables, multivariate ANOVA was utilized to assess the differences between the groups. *p* < 0.05 indicated that a difference was statistically significant. GraphPad Prism 5.0 was employed for drawing plots.

## Results

### Acquisition of Potential Active Ingredients in GGQLD and Common Targets of HUA

**FIGURE 1:**
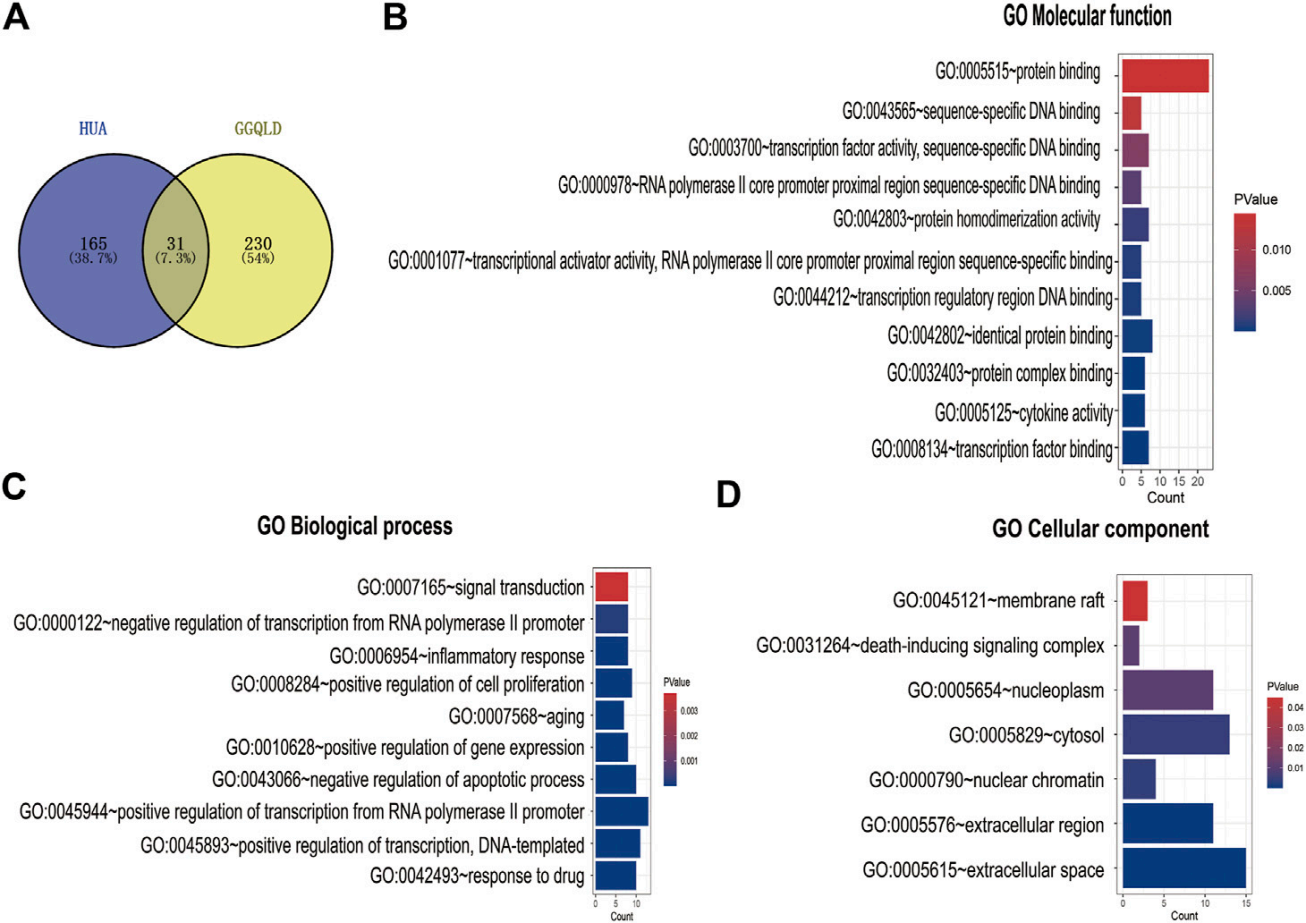
GO enrichment analysis of the common targets. **(A)** The Venn diagram of both GGQLD targets and HUA targets. **(B)** GO molecular function analysis of overlapping targets. **(C)** GO biological process analysis of overlapping targets. **(D)** GO cellular component analysis of overlapping targets.

A total of 907 ingredients were discovered in GGQLD through the TCMSP database, including 62, 123, 178, and 278 in Gegen, Huangqin, Huanglian, and Gancao, respectively. The thresholds of DL ≥ 0.18 and OB ≥ 30% were used to screen the active ingredients. Finally, 372 candidate active ingredients conformed to our preset screening thresholds. A total of 196 genes in HUA were screened from the database (DisGeNET). In TCM, a medication displays diverse pharmacological effects via various targets. Therefore, it is greatly significant to determine the TCM medication mechanisms in treating complicated diseases based on network analysis. Previously, we acquired a total of 196 targets for HUA from the DisGeNET database. Then, targets were predicted by incorporating 372 active ingredients in the UniProt database. Consequently, 31 overlapped targets between GGQLD and HUA were obtained by VENN map (Figure 1A).

### Potential Therapeutic Mechanisms of GGQLD in HUA

**FIGURE 2:**
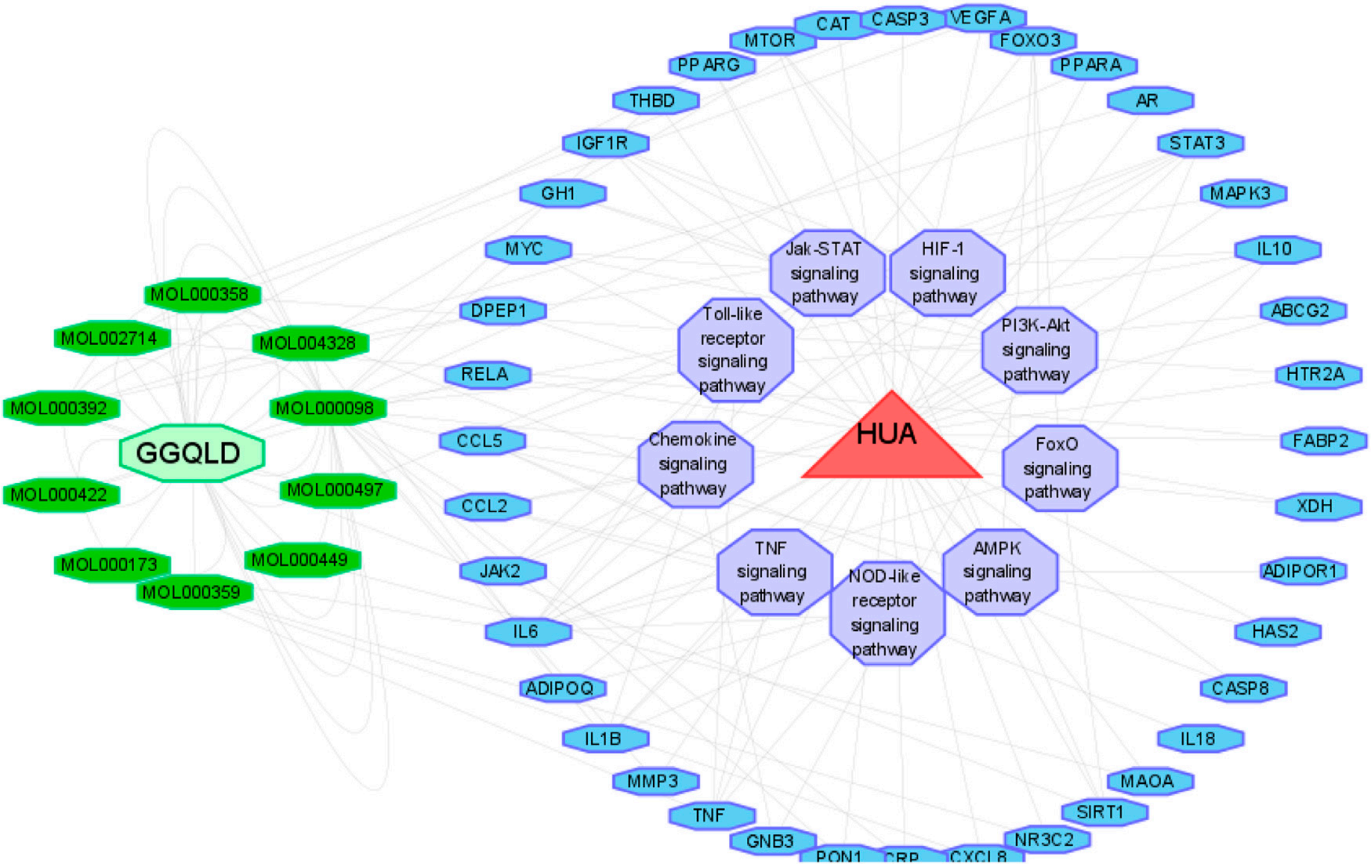
Potential therapeutic mechanisms of GGQLD in hyperuricemia. **(A)** Bubble chart of the top 35 signaling pathways screened by using the KEGG enrichment analysis. **(B)** Venn diagram showing the overlap genes between five types of pathways. **(C)** Protein–protein interaction and gene coexpression network.

**FIGURE 3:**
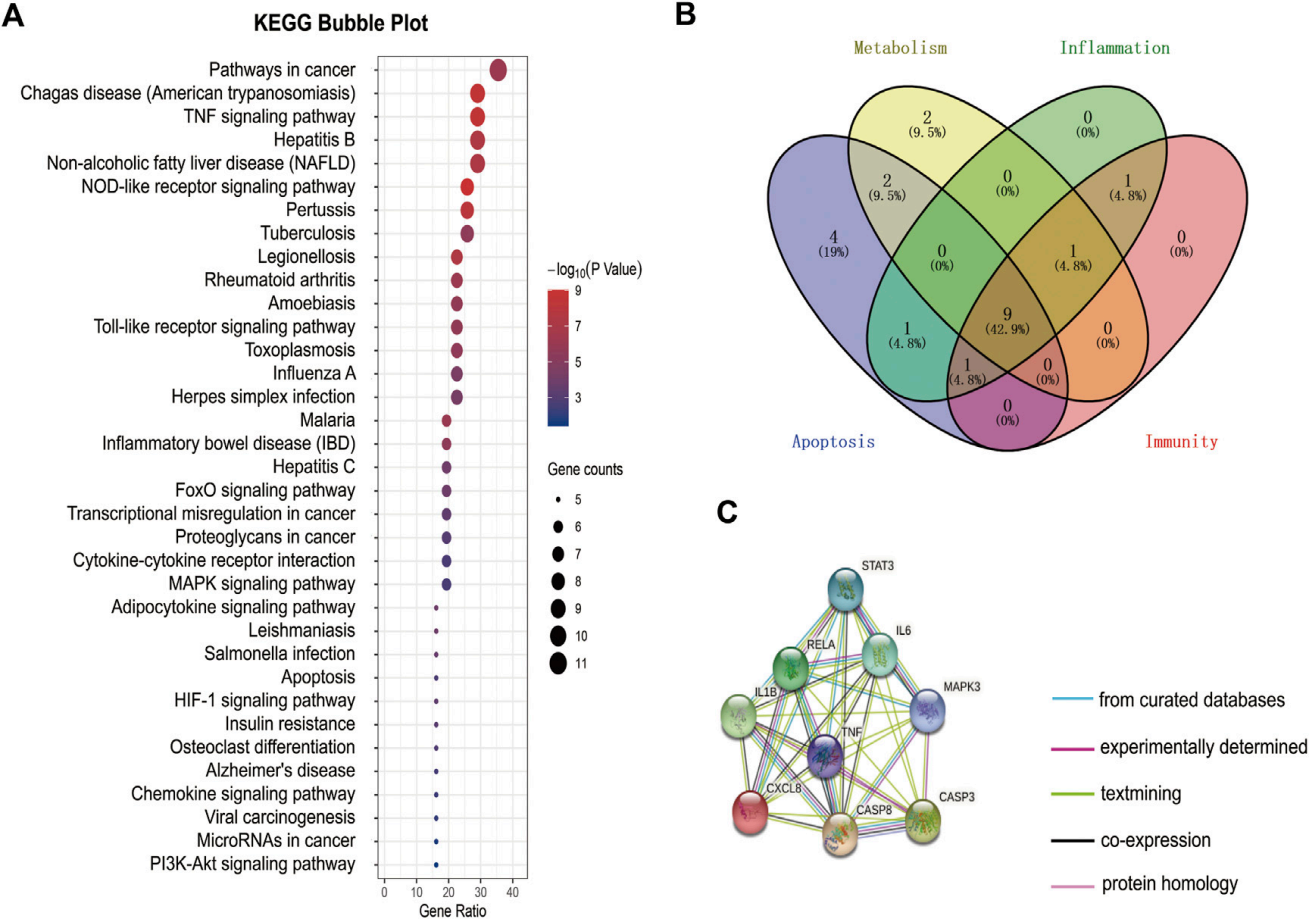
Drug–compound–target–disease network map.

We performed GO functional annotations of the 31 identified genes, suggesting their involvements in cell components (CCs), biological processes (BPs), and molecular functions (MFs). To be specific, these genes were mainly involved in various BPs, including inflammatory response and negative regulation of the apoptotic process (Figures 1B–D). In addition, according to KEGG pathway analysis, the intersected genes were mainly associated with 40 pathways. Among them, 35 pathways with the most significant *p*-values are shown in Figure 2A including cancers, apoptosis, and inflammatory signaling pathways. In addition, we also constructed the active ingredient–target–disease network by adopting Cytoscape 3.8.0 software, obtaining the interactions among drugs, compounds, diseases, and targets. The results are shown in Figure 3. Obviously, 17 targets were related to the apoptosis pathways, 14 to the metabolism pathways, 13 to the inflammation pathways, 13 to the immunity pathways, and 14 to the signal transduction pathways. As illustrated by the VENN map, we acquired 8 intersected targets in the 5 pathways (Figure 2B). Moreover, the STRING platform was adopted to establish the protein–protein interaction (PPI) (Figure 2C).

### GGQLD Downregulated the Expression of NLPR3 in PBMCs and Urinary Exosomes in HUA Patients

**FIGURE 4:**
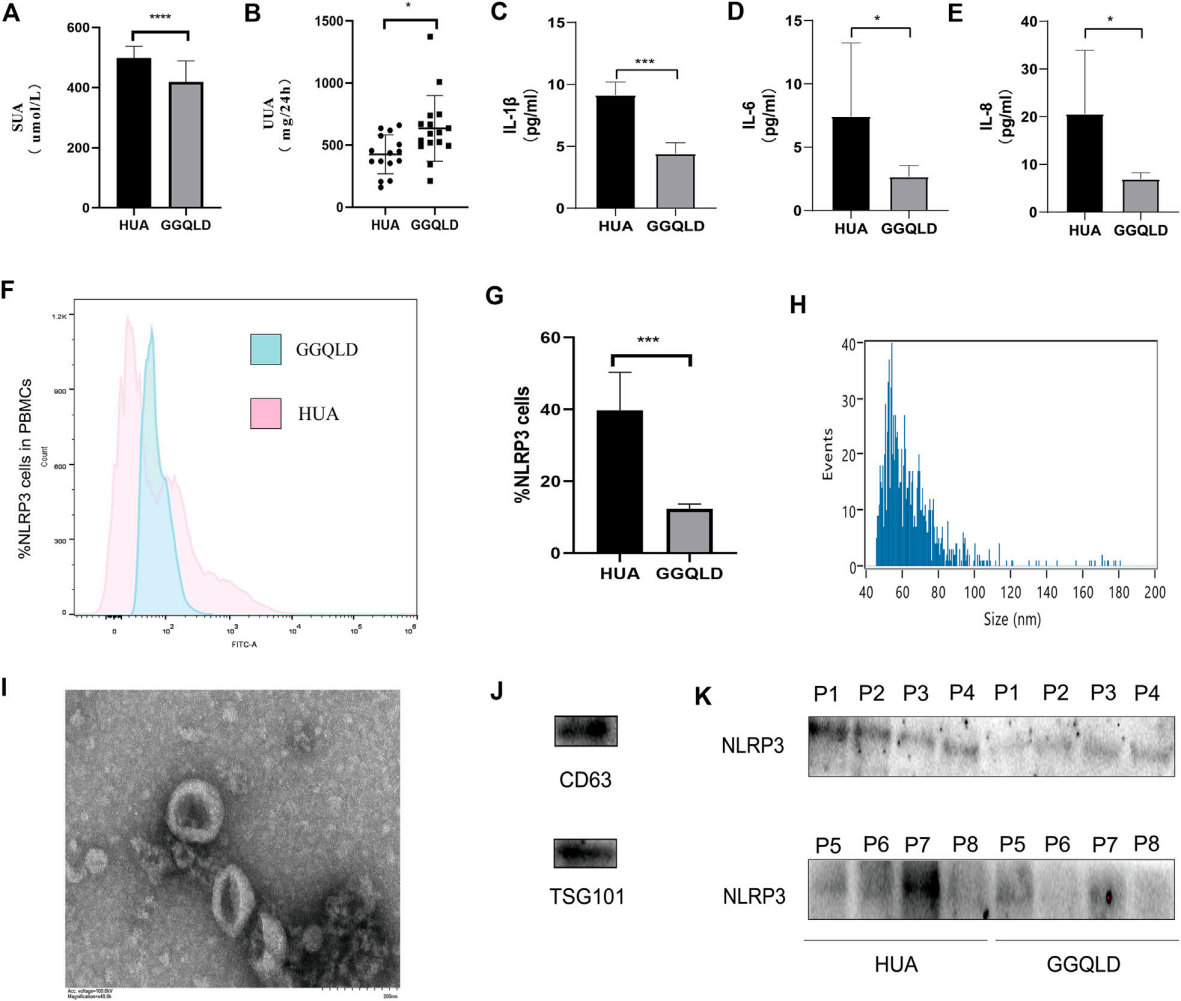
NLRP3 expression is suppressed in HUA patients after GGQLD treatment. **(A–E)** Serum uric acid, 24 h urine uric acid, IL-1β, IL-6, and IL-8 were studied before and after medication. **(F)** PBMCs, freshly isolated by Ficoll–Paque density gradient centrifugation, were stained with mAbs conjugated with FITC and data acquired by flow cytometry. Cells were gated as shown in Figure. **(G)** The expression of NLRP3 inflammatory protein in PBMCs of HUA patients was significantly reduced after GGQLD treatment. **(H–J)** Exosome nanoparticle tracking analysis; Exosomes under electron microscope; the characterization exosomes with related markers. **(K)** The expression of NLRP3 inflammatory protein in urinary exosomes of HUA patients was significantly reduced after GGQLD treatment. For **(K)**, *n* = 3 independent experiments per group. In all statistical plots, data are expressed as the mean ± SD, *p < 0.05, **p < 0.01, ***p < 0.001, ****p < 0.0001.

We found that the SUA and UUA levels (Figures 4A,B) in HUA patients were reduced after GGQLD treatment. Besides, the levels of, IL-6, and IL-8 (Figures 4C–E) also significantly decreased in the GGQLD group. According to our previous study, soluble UA induced NLRP3 inflammasome production. To define the possible therapeutic approaches of HUA-induced renal tubular injury, the present study analyzed the expression of NLRP3 in PBMCs and that of urinary exosome protein in HUA patients before and after GGQLD treatment. By isolating PBMCs and extracting urinary exosomes from HUA patients before and after treatment, this study conducted flow cytometry and WB assays to analyze two groups of PBMCs and urinary exosomes. As a result, GGQLD downregulated the expression of NLRP3 in HUA patients (Figure 4F,G,K).

### HPLC Profiles of GGQLD and Its Fractions

We obtained GGQLD extract through the purification of chromatographic grade methanol, since saponin and flavone were the major bioactive ingredients in GGQLD. Besides, HPLC analysis was performed to investigate the chemical features of GGQLD extract, with methanol being the eluent. To obtain the superb efficiency and favorable separation selectivity of HPLC analysis, we optimized the composition of the mobile phase (10 mmol/l acetonitrile–water contained within the ammonium formate solution). According to Supplementary Figure S1, the major bioactive ingredient puerarin was identified in Gegen, baicalin in Huangqin, berberine in Huanglian, and liquiritin in Gancao, which were later adopted to construct a combined standard approach. Accordingly, we detected four peaks of GQT extract from the HPLC chromatogram and adopted related chemical standards to quantify levels of puerarin, liquiritin, berberine, and baicalin (Peaks A–D), respectively. To be specific, the contents of the above four active ingredients were 3.9, 0.3, 2.9, and 1.0 mg/ml in GGQLD extract, respectively (Table 3).

**TABLE 3:** Contents of the four major compounds in GGQLD.

Sample name	Puerarin (mg/ml)	Liquiritin (mg/ml)	Baicalin (mg/ml)	Berberine (mg/ml)
Sample 1	3.971	0.310	2.970	1.047
Sample 2	3.967	0.312	2.983	1.051
Sample 3	3.969	0.306	2.967	1.048

### GGQLD Alleviated HUA-Induced Renal Tubular Inflammation and Apoptosis *In Vivo*

**FIGURE 5:**
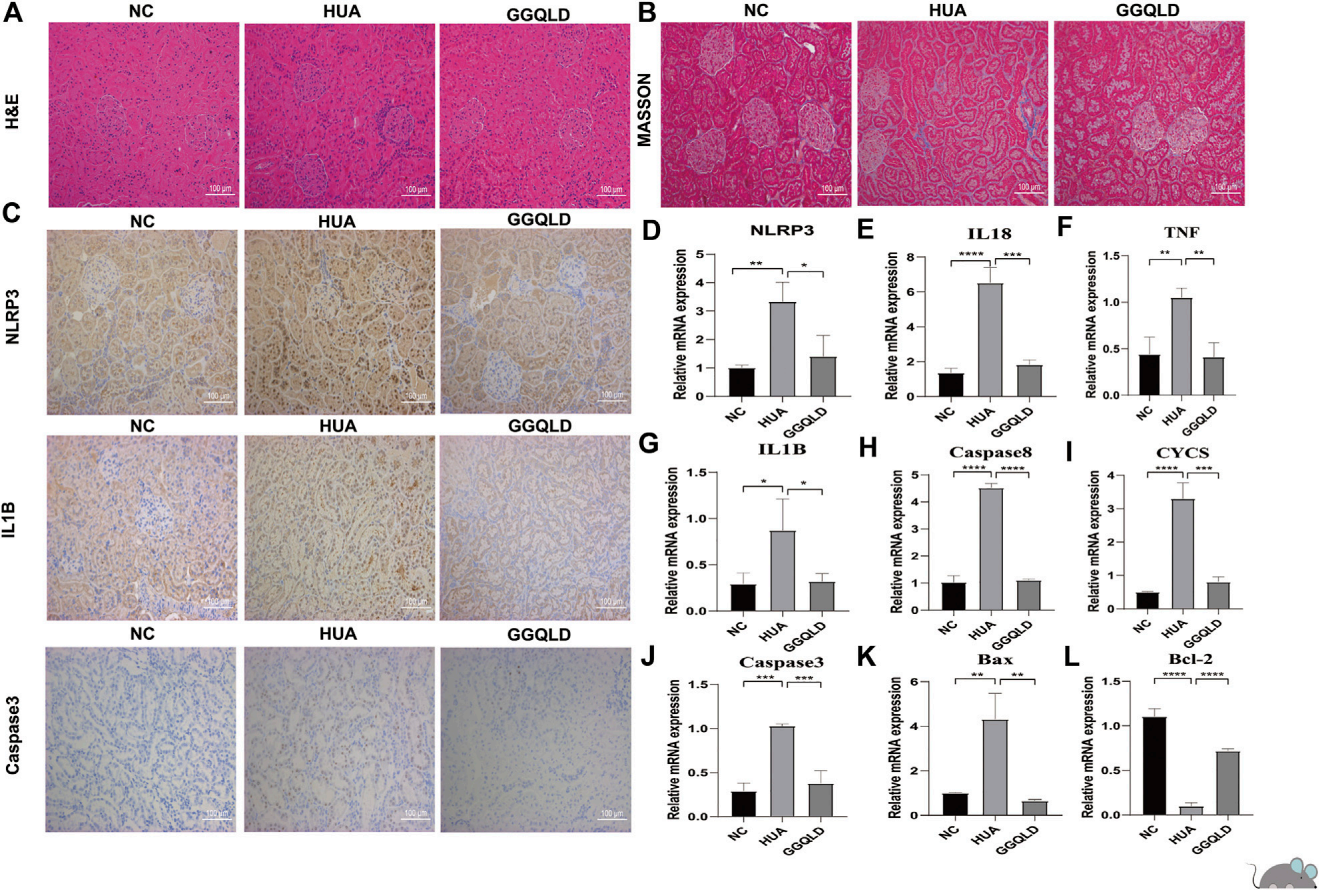
GGQLD therapy inhibits NLRP3 expression and expression of the apoptosis gene *in vivo*. **(A)** The renal histological injury of three groups was measured by HE staining. **(B)** The renal fibrosis of four groups was determined by Masson trichrome staining. **(C)** Representative immunohistochemical staining images of NLRP3, IL1β, and caspase-3 expression in renal tissue. *N* = 3 rats/group. Scale bars, 100 mm. **(D,E,G)** GGQLD protected the kidney through suppressing the relative mRNA levels of NLRP3 inflammasomes, IL-1β, and IL-18 in HUA rats. **(F,H–L)** GGQLD alleviated the relative mRNA levels of apoptosis-related genes caspase-3, caspase-8, and TNF and simultaneously alleviated the mRNA levels of mitochondrial apoptosis-related genes Bax, Bcl-2, and CYCS in HUA rats. For **(D–L)**, *n* = 3 independent experiments per group. In all statistical plots, data are expressed as the mean ± SD, *p < 0.05, **p < 0.01, ***p < 0.001, ****p < 0.0001.

Furthermore, the current work verified the protection of GGQLD against HUA in rat models. In brief, gastric OA at 750 mg/kg/day was given to male SD rats for eight consecutive weeks. Meanwhile, GGQLD at 10 ml/kg/day initiating on week 5 was given for four consecutive weeks. As a result, NLRP3 staining and mRNA expression increased in renal cortical tissues of OA rats (Figures 5C,D). GGQLD reduced the NLRP3 expression in OA-treated rats (Figures 5C,D). GGQLD significantly attenuated the mRNA expression and staining of IL-1β and caspase-3 in renal tissues of UA rats (Figures 5C,G,J). Additionally, it was found that GGQLD significantly alleviated the mRNA expression of TNF, caspase-3, caspase-8, Bax, Bcl-2, and CYCS in renal tissues of HUA rats (Figures 5F,H–L).

### GGQLD Inhibited Mitochondrial Apoptosis and Inflammation of PTECs

**FIGURE 6:**
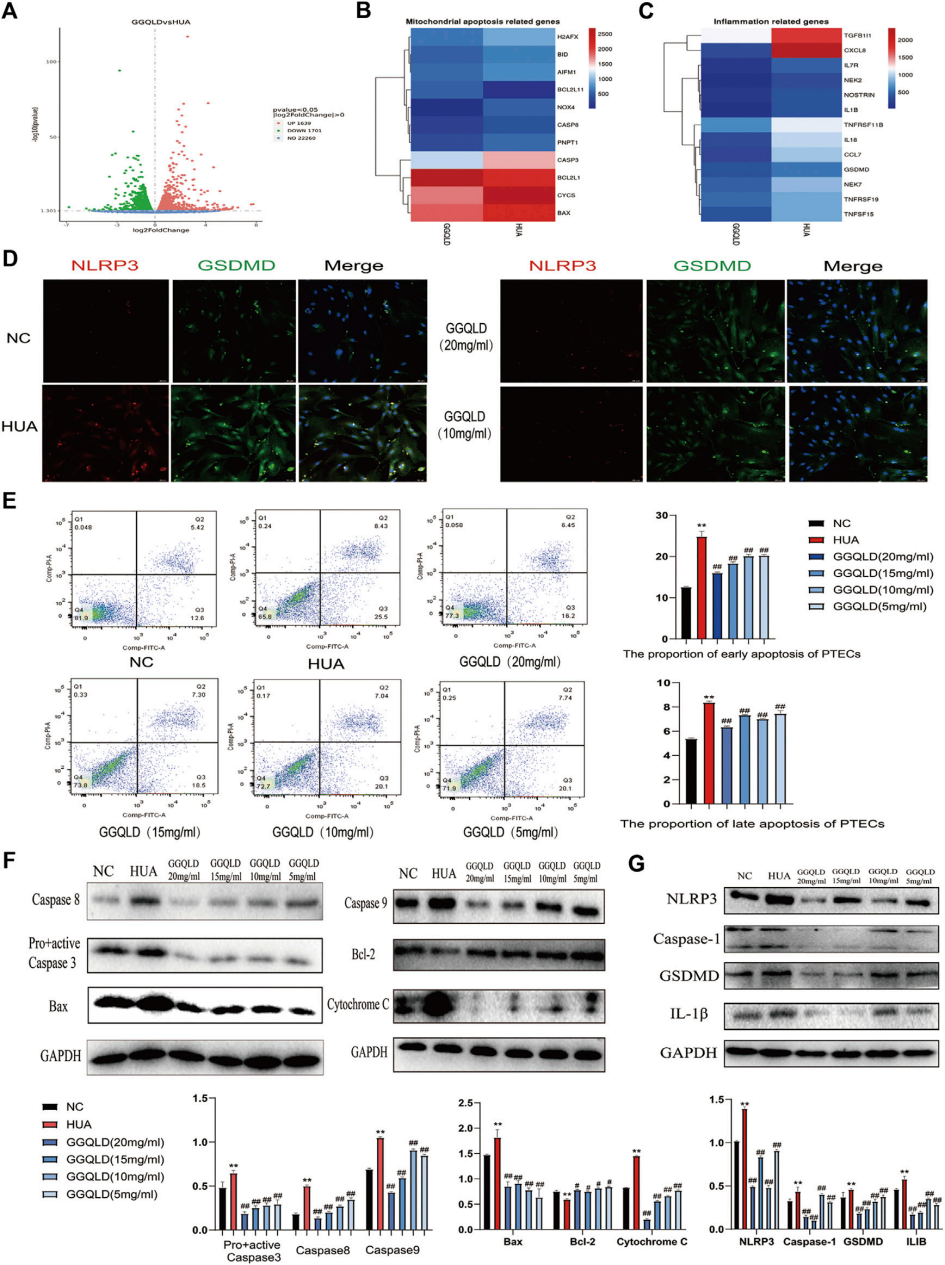
GGQLD inhibits mitochondrial apoptosis and inflammation in PTECs. **(A)** Gene expression profile was compared between cells in response to GGQLD treatment, and heat maps were generated based on expression of the significantly changed genes related to inflammatory response and the mitochondrial apoptosis process **(B,C)**. **(D)** GGQLD alleviating inflammasome-induced pyroptosis through inhibiting the NLRP3/GSDMD signal in UA-stimulated PTECs. **(E)** GGQLD alleviated early and late apoptosis. **(F,G)** GGQLD also reduced Bcl-2, Bax, cytochrome c, pro + active caspase-3, cleaved caspase-3, caspase-9, and caspase-8 expression in UA-stimulated PTECs. The levels of key proteins of the NLRP3 signaling cascade in PTECs by GGQLD treatment. For **(D–G)**, *n* = 3 independent experiments per group. *p < 0.05 vs. NC, **p < 0.01 vs. NC, #*p* < 0.05 vs. HUA, ##*p* < 0.01 vs. HUA.

According to the CCK-8 results, different doses of GGQLD (20, 15, 10, and 5) were used to treat the UA-induced PTECs (Supplementary Figure S2). Based on our RNA-seq data, GGQLD was found to suppress BPs related to mitochondrial apoptosis and inflammatory response (Figures 6A–C). Moreover, GGQLD significantly reduced the expression of NLRP3 and GSDMD compared with UA (Figure 6D). UA remarkably promoted apoptosis at the early and late stages (Figure 6E), while GGQLD had the opposite effect (Figure 6E). Besides, we also investigated the effect of GGQLD on the UA-induced expression of Bcl-2 and caspase gene family proteins in PTECs. Consequently, as further confirmed by WB assays, GGQLD significantly reduced the UA-induced NLRP3 expression (Figures 6F,G).

### GGQLD Down-Regulated the Expression of Urate Transporter in UA-Stimulated PTECs

**FIGURE 7:**
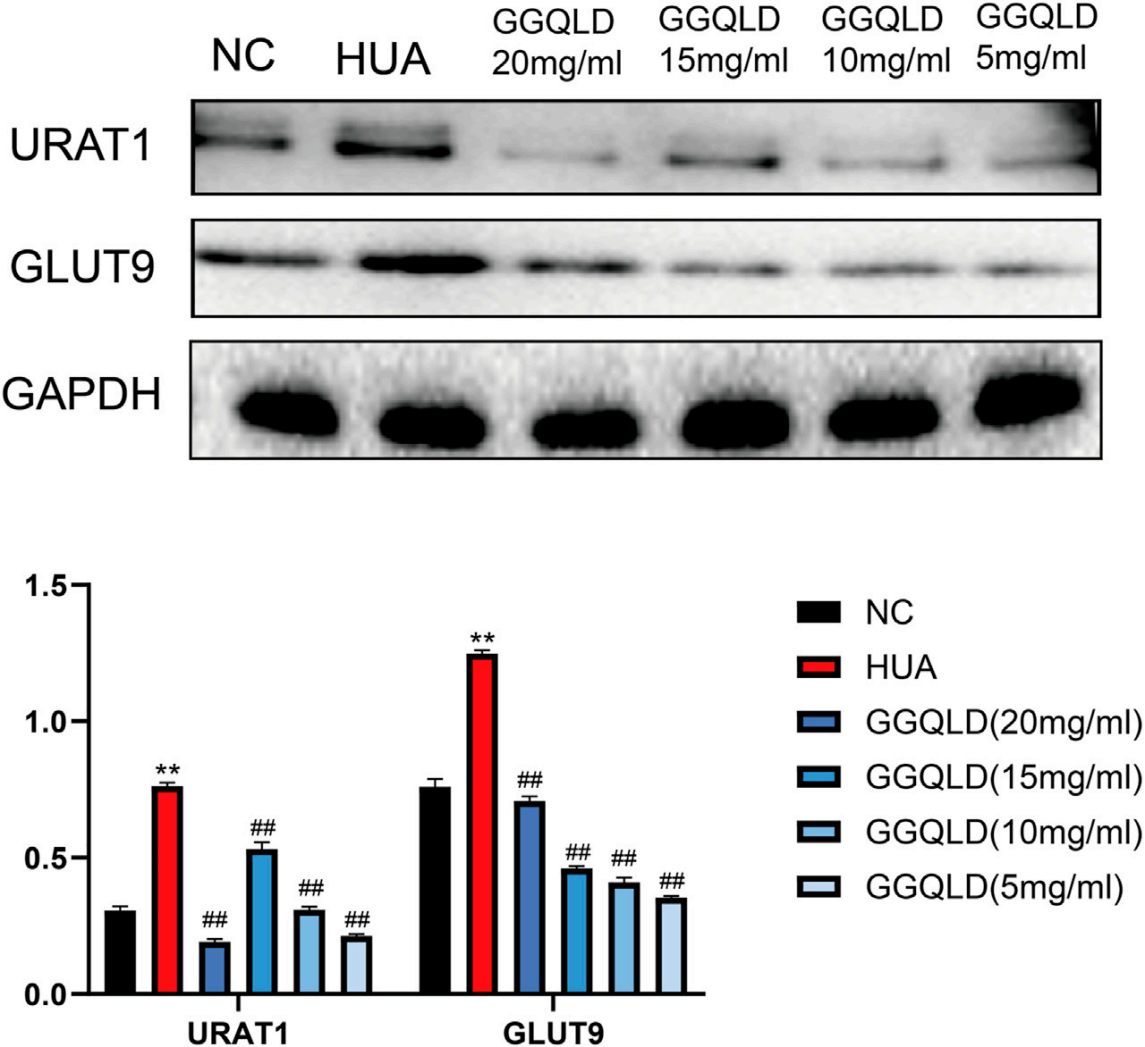
GGQLD alleviated urate transporter expression in UA-stimulated PTECs. GGQLD significantly reduced URAT1 and GLUT9 expressions. *n* = 3 independent experiments per group. *p<0.05 vs. NC, **p < 0.01 vs. NC, #*p* < 0.05 vs. HUA, ##*p* < 0.01 vs. HUA.

UA is mainly regulated by urate transporters. To explore the effect of GGQLD on HUA, this study investigated its effects on URAT1 and GLUT9. As a result, GGQLD significantly downregulated the expression of URAT1 and GLUT9 (Figure 7) in comparison with the HUA group.

**FIGURE 8:**
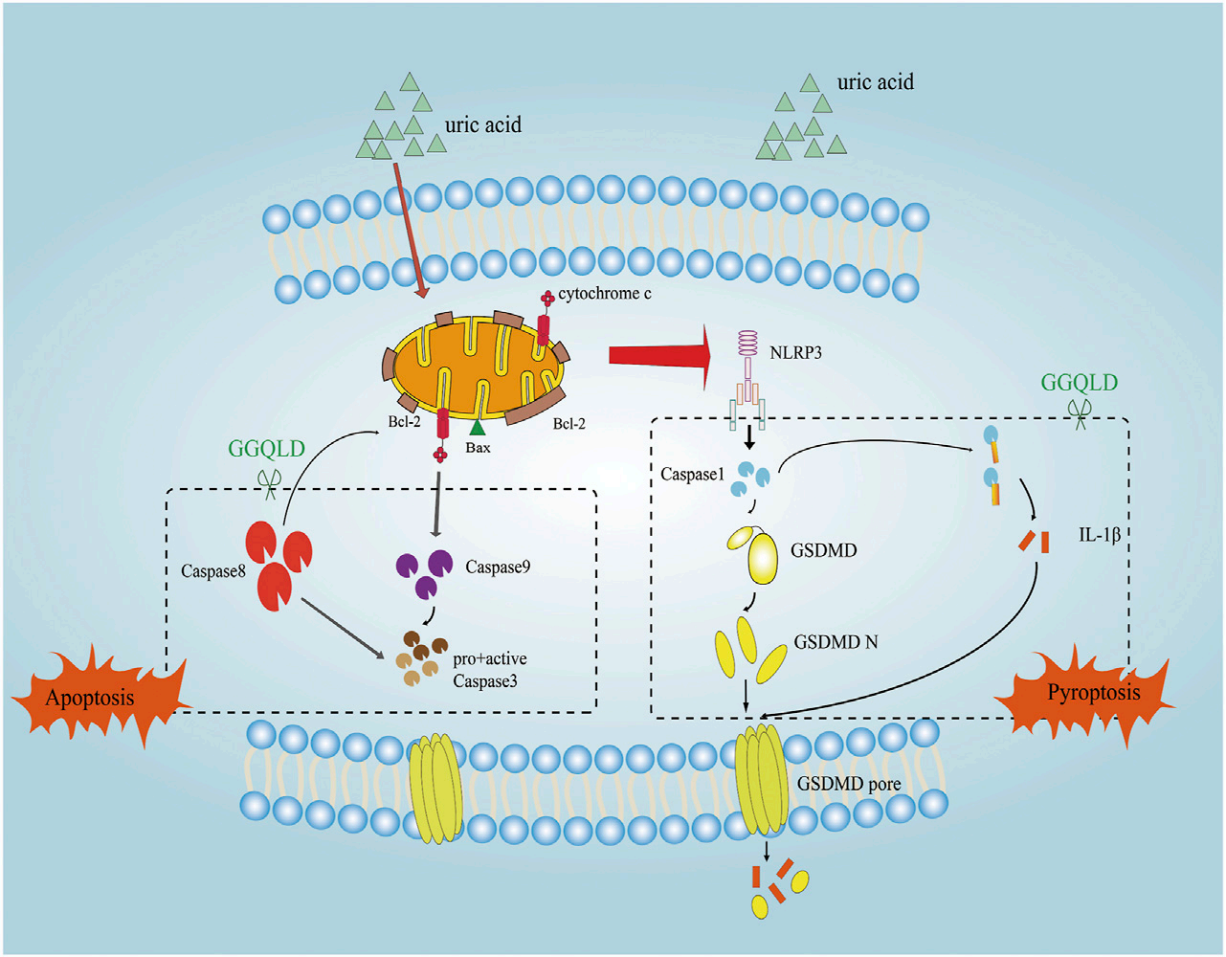
The mechanism of the protective effect of GGQLD in HUA-induced renal injury. The increased UA led to PTEC mitochondrial apoptosis and assembled NLRP3 inflammasomes and facilitated IL-1β maturation and thus inflamed the cells, which damaged the mitochondria and caused pyroptosis to lead to renal tubule damage. GGQLD protected cells from inhibiting the mitochondrial apoptotic pathways through capsase-9, caspase-8, and Bcl-2/Bax/caspase-3, thus alleviating inflammation via inhibition of NLRP3/caspase-1/IL-1β/GSDMD.

## Discussion

In the present study, clinical data proved that GGQLD reduced the SUA levels and NLRP3 expression in PBMCs and urinary exosomes in asymptomatic HUA patients. In addition, it could also be demonstrated that GGQLD significantly suppressed NLRP3 inflammasome production within the renal cortical tissues in HUA rats and UA-stimulated PTECs. GGQLD significantly prevented the UA-induced renal inflammation, which caused apoptosis via the NLRP3 signaling pathway and the mitochondrial-dependent pathway. The HUA-induced renal injury was significantly alleviated by GGQLD *in vitro* and *in vivo*.

It is beneficial to inhibit receptors and inflammasomes to reduce renal fibrosis and inflammation. Knocking down Aim2 and NLRP3 can alleviate renal fibrosis, inflammation, and injury (Kopalli et al., 2016; Cao et al., 2021). In clinical settings, GGQLD has frequently been adopted for the treatment of diabetes and UC (Xu et al., 2011; Zhao et al., 2020). GGQLD has been suggested to suppress the inflammatory signal transduction pathway and promote the antioxidant effect, thus improving UC. Additionally, GGQLD promotes glucose metabolic disorders, protects pancreatic β cells, and elevates the insulin sensitivity index, thereby exerting an important role in treating diabetes (Ahmed et al., 2017). On this basis, we performed bioinformatics analysis to identify potential therapeutic targets of GGQLD. Our results of the systemic pharmacological analysis proved that the treatment of GGQLD mainly involved the anti-inflammatory and antiapoptotic pathways. In our previous study, NLRP3 was found to be related to the pathogenesis of HUA. Therefore, we hypothesized that GGQLD targeted NLRP3 to exert its anti-inflammatory activity.

NLRP3 is one of the key elements during the activation of inflammation, which can interact with an apoptotic speck-like protein that contains a caspase recruitment domain (CARD) (ASC) through the pyrin domain (PYD). Later, ASC will recruit and activate pro-caspase-1 via CARD. The aforementioned interaction constitutes the NLRP3 inflammasome, which is a kind of great cytosolic protein complex (Csak et al., 2011; Wree et al., 2014; Shi et al., 2015; Abraham et al., 2016). Afterward, the activation of NLRP3 inflammasomes can activate caspase-1. First, caspase-1 cleaves gasdermin D (GSDMD), which can be activated for releasing the active N-terminal protein, and the latter can mediate pyroptosis (Derangère et al., 2014; Ding et al., 2016; Sborgi et al., 2016; Liu et al., 2017). Second, the activation of caspase-1 will recruit and activate the inflammatory molecules like IL-1β, while inducing inflammatory responses. Pyroptosis, also called cellular inflammatory necrosis, can promote the release of cellular contents to activate the inflammatory response (Shi et al., 2017; Wang et al., 2018). Evidence from previous studies shows that the NLRP3 inflammasome-induced GSDMD-dependent pyroptosis, accompanied by IL-1β processing, is responsible for renal tubular epithelial cell injury (Miao et al., 2019; Wang et al., 2019; Yasukawa and Koshiba, 2021). In our study, we first compared the difference of NLRP3 expression in PBMCs and urinary exosomes of HUA patients before and after treatment, finding that the expression of NLRP3 significantly decreased after treatment, providing that GGQLD exhibited significant anti-inflammation activity. We further validated and demonstrated that GGQLD significantly reduced the expression of NLRP3, caspase-1, GSDMD, and IL-1β in the UA-stimulated PTEC model *in vitro*, suggesting that GGQLD alleviated IL-1β processing and the subsequent amplification of inflammatory cascades. Some scholars have proved that GGQLD exerts an anti-inflammatory effect, and we confirmed that GGQLD showed anti-inflammatory effect by targeting NLRP3-induced GSDMD-dependent pyroptosis in the treatment of HUA.

The mitochondrion is an organelle with multiple functions, which is involved in a lot of biological processes, such as energy metabolism and cell suicide (Dagvadorj et al., 2021). It is currently proposed that mitochondrial damage accounts for an important determining factor for the activation of NLRP3 inflammasomes (Mohan et al., 2012). The NLRP3 stimuli–induced mitochondrial destruction contributes to exposing mitochondrial DNA (mtDNA) into cytoplasm and generating reactive oxygen species (ROS). The mtDNA in cytoplasm can colocalize with NLRP3, which can thus promote IL-1β release, whereas the oxidized mtDNA can serve as the stronger factor to induce IL-1β secretion. Till the present, mtDNA is only associated with the activation signal related to the responses of NLRP3 inflammasomes. According to the network pharmacology analysis, it is considered that GGQLD exhibited protective effects on uric acid–induced renal injury through the apoptotic pathway. Therefore, we speculated that GGQLD may protect HUA-induced renal injury through the antimitochondrial apoptosis pathway.

Proteins in the Bcl-2 family, which include the proapoptotic proteins (like Bad, Bal, and Bax) and the antiapoptotic proteins (like Bcl-2, Mcl-1, and Bcl-xl), are responsible for regulating mitochondrial disruption (Degenhardt et al., 2002; Vucicevic et al., 2016; Peña-Blanco and García-Sáez, 2018). Among them, the proapoptotic proteins play the roles of the mitochondrial pathway promoters. After being stimulated, Bak and Bax will be transported in the mitochondrial membrane, which increases the permeability of the mitochondrial outer membrane, causing caspase cascade activation and cytochrome c release (Dairaku et al., 2004; Fang et al., 2012). In addition, the antiapoptotic proteins play the roles of suppressors through suppressing cytochrome c release (Mo et al., 2019). Bcl-2 is reported to directly reduce mitochondrial membrane permeability through binding to the mitochondrial outer membrane protein voltage-dependent anion channel 1 (VDAC1). Therefore, inducing Bax transport in mitochondria and inhibiting Bcl-2 transport in mitochondria can serve as two approaches for inducing the mitochondria-regulated apoptosis. In our study, we found that GGQLD significantly attenuated the miRNA expression of caspase-3, caspase-8, Bax, Bcl-2, and CYCS in renal tissue from HUA rats. We also examined the effect of GGQLD on the UA-induced expression of Bcl-2 and caspase gene family proteins in PTECs. It can be demonstrated that GGQLD can protect HUA-induced renal epithelial cells by inhibiting mitochondrial apoptosis.

In summary, GGQLD positively affects HUA by suppressing apoptosis and enhancing renal inflammation, which provides laboratory data supporting its clinical application. All the medicinal herbs in GGQLD have been utilized in TCM for thousands of years. Therefore, GGQLD is regarded to be safe and tolerable. GGQLD treatment reduces IL-1β production, NLRP3, caspase-1, and GSDMD expression depending on its concentration. In addition, GGQLD inhibits the apoptosis of PTECs, upregulates the expression of Bcl-2, and downregulates that of Bax, cytochrome c, and caspase-3/8/9. The present work suggests that GGQLD is the best treatment for HUA, which exerts its effects through suppressing apoptosis and renal inflammation. More studies are warranted to examine the systemic molecular mechanisms of GGQLD via the selection of diverse blockers and development of experimental technologies.

## Data Availability

The data presented in the study are deposited in the ENA repository, accession number is PRJEB43968.
