# Supplementary material for: Gegen Qinlian Decoction Ameliorates Hyperuricemia-Induced Renal Tubular Injury via Blocking the Inflammatory Signaling Pathway
Source: Front Pharmacol. 2021 May 4;12:665398. doi: 10.3389/fphar.2021.665398 (PMC8129546; doi:10.3389/fphar.2021.665398)

**Supplements**

**Supplement 1: HPLC analysis of three batches of GGQLD aqueous preparation.**

Contents of chemical markers of GGQLD aqueous preparation (Batch A is the sample used in the experiment). (A) HPLC chromatogram of standard reference; (B) HPLC chromatogram of Batch A; (C) HPLC chromatogram of Batch B; and (D) HPLC chromatogram of Batch C.


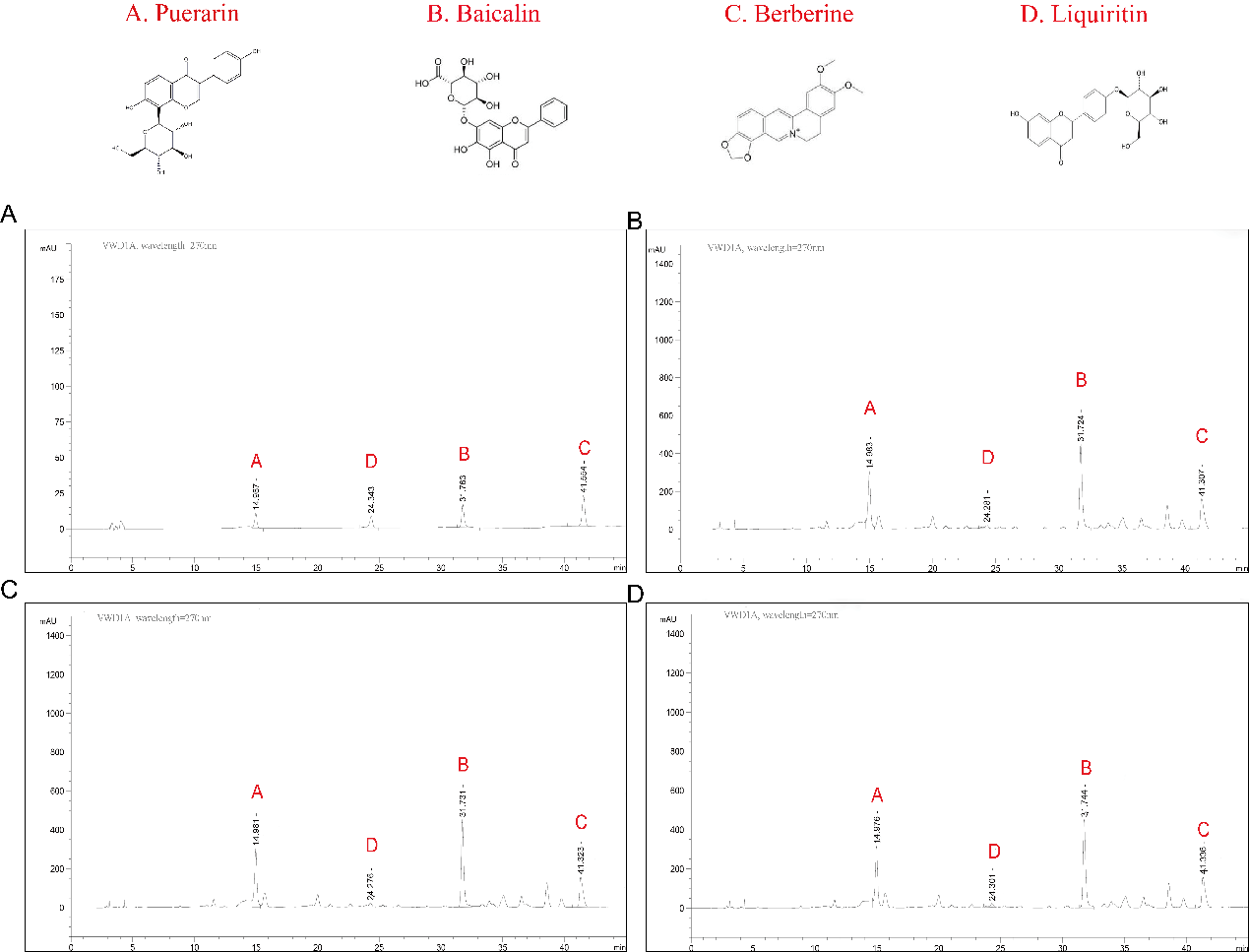


**Supplement 2: Cell viability were performed by Cell Counting Kit-8 experiment.**


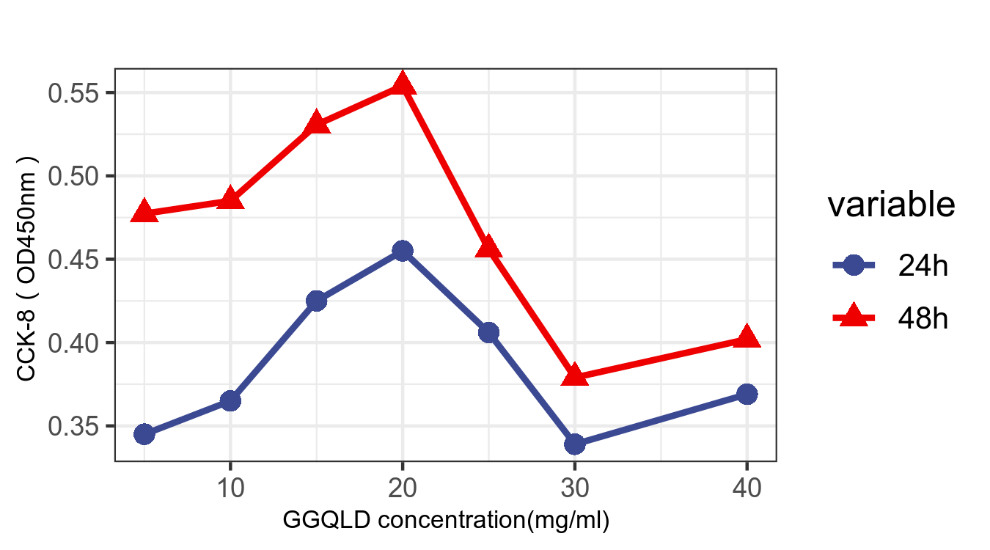

Supplement: Supplementary file 1 [file DataSheet1.docx]
